# Progress in Global Surgery: Comment on "Global Surgery – Informing National Strategies for Scaling Up Surgery in Sub-Saharan Africa"

**DOI:** 10.15171/ijhpm.2018.69

**Published:** 2018-08-08

**Authors:** Haile T. Debas

**Affiliations:** Institute for Global Health Sciences, University of California, San Francisco, CA, USA.

**Keywords:** Global Surgery, Universal Health Coverage, District Hospital

## Abstract

Impressive progress has been made in global surgery in the past 10 years, and now serious and evidence-based national strategies are being developed for scaling-up surgical services in sub-Saharan Africa. Key to achieving this goal requires developing a realistic country-based estimate of burden of surgical disease, developing an accurate estimate of existing need, developing methods, rigorously planning and implementing the plan, and scaling-up essential surgical services at the national level.

The Editorial Opinion by Gajewski et al^1^ illustrates how much progress has been made in global surgery in recent years. We have come a long way in the past 10 years when the major effort was to have global surgery be considered as an important component of global public health. Contrast this to the main focus of the editorial by Gajewski et al on “the development of national surgical plans and the research agenda for informing the development, implementation and sustainable scale-up of surgical systems.”

An inflection point in the appreciation of the importance of global surgery and its key role in the implementation of universal health coverage occurred in 2015 with the publication of the Lancet Commission on Global Surgery^2^ and Disease Control Priorities, Third Edition, Volume 1: Essential Surgery.^3^ These two publications have elevated the discourse on essential surgery and pointed the way forward to plan and implement the global surgery agenda.

In my view, there are four critical steps in the implementation of a global surgery agenda: (*a*) developing a realistic estimate of the burden of surgical diseases in each country; (*b*) assessing the actual gap in provision of essential surgery care in each country; (*c*) developing a national strategy for implementation of a plan that specifically addresses the service gap; and (*d*) the actual implementation of the services and a plan for their national scale-up.

Most estimates of the burden of surgical disease are based on expert opinion and modelling. Yet, what we need is realistic estimates contextualized to each individual country so that more focused and highly relevant plans can be made for the implementation of essential surgical services. In this regard, the Lancet Commission on Global Surgery has provided the best approach so far, combining reviews of national level data with interviews of local surgical specialists and Ministries of Health. This approach also is likely to give the most accurate assessment of the gap in the provision of surgical services. In either case, however, urgent need for implementation research exists, as pointed out by Gajewski et al in their editorial opinion.^1^

Many well-meaning global surgery initiatives in sub-Saharan Africa fail to achieve their goals because they do not partner with local Ministries of Health and lack sustainability. Without the active participation of the Ministries of Health, no strategy for a national plan or its implementation is possible. In many sub-Saharan countries, the regional allocation of the annual budget does not include a line item for surgery. Ministries of Finance allocate budgets, so advocacy efforts should be made to both Ministries of Health and Finance.

The first-level or district hospital plays a pivotal role in providing essential surgery care. It is the most cost-effective surgical platform with $10-220 per disability-adjusted life year (DALY) averted for surgical care delivered in it.^4^ In any case, much of the implementation research that needs to be done must recognize the central role the first-level hospital.

Finally, the case study of Zambia, described by Gajewski et al,^1^ demonstrates the key ingredients to success: the central role of the district hospital, the importance of collaboration between a US academic institution and the Ministry of Health and local surgical champions, and multi-lateral sources of funding. It is clear that significant progress is being made in implementing the essential surgery care agenda, but this is just the beginning. Bilateral and multilateral organizations and other global donors must recognize that without making essential surgery care accessible, safe and affordable, the lofty ideal of universal health coverage cannot be realized.

## Ethical issues

Not applicable.

## Competing interests

Author declares that he has no competing interests.

## Author’s contribution

HTD is the single author of the paper.
